# Correction: Epidemiological factors associated with immunological resistance in household contacts exposed to active tuberculosis in South Africa: A logistic regression analysis

**DOI:** 10.1371/journal.pone.0337706

**Published:** 2025-11-25

**Authors:** Nomfanelo Maenetje, Matthew Oladimeji, Mandla Mlotshwa, Matt A Price, Daniel Hoft, Christina Lindan, Robert Wallis, Salome Charalambous, Gavin Churchyard, Vinodh Edward, Andrew Fiore-Gartland, Jerry Shai, Pholo Maenetje

Matt A Price should be included in the author byline. Matt A Price should be listed as the fourth author, and his affiliation is 6: Department of Epidemiology and Biostatistics, University of California at San Francisco, San Francisco, California, United States of America. The contributions of this author are as follows: Analyze or synthesize study data, Supervised oversight and leadership responsibility for the research, Wrote/ reviewed & edited the paper.

The correct citation is: Maenetje N, Oladimeji M, Mlotshwa M, Price MA, Hoft D, Lindan C, et al. (2025) Epidemiological factors associated with immunological resistance in household contacts exposed to active tuberculosis in South Africa: A logistic regression analysis. PLoS One 20(8): e0329562. https://doi.org/10.1371/journal.pone.0329562
