# Supplementary material for: Epidemiological factors associated with immunological resistance in household contacts exposed to active tuberculosis in South Africa: A logistic regression analysis
Source: PLoS One. 2025 Aug 19;20(8):e0329562. doi: 10.1371/journal.pone.0329562 (PMC12364365; doi:10.1371/journal.pone.0329562)
Supplement: S1 Table — (DOCX) [file pone.0329562.s001.docx]

**Suppl. Table 1. Factors associated with being QFT-TST- amongst household contacts stratified by risk score**

|  | **Restricted to high risk HHCs (n=91)** | | | | | | | **Restricted to low risk HHCs (n=64)** | | | | | | |
| --- | --- | --- | --- | --- | --- | --- | --- | --- | --- | --- | --- | --- | --- | --- |
| **Variable** | **Total** | **QFT-/TST-** | | **Univariate**  **analysis** | | **Multivariate analysis** | | **Total** | **QFT-/TST-** | | **Univariate**  **analysis** | | **Multivariate**  **analysis** | |
|  | **N** | **n** | **%** | **Unadjusted OR (95% CI)** | **p-value** | **Adjusted OR (95% CI)** | **p-value** | **N** | **n** | **%** | **Unadjusted OR (95% CI)** | **p-value** | **Adjusted OR (95% CI)** | **p-value** |
| **Age, years** |  |  |  |  |  |  |  |  |  |  |  |  |  |  |
| 18-30 | 37 | 13 | **0.35** | 1.68 (0.65-4.37) | 0.3 |  |  | 9 | 3 | **0.33** | 9.34 (1.12-78.16) | 0.04 | 9.34(1.12-78.16) | **0.04** |
| 31-40 | 14 | 3 | **0.21** | 0.89 (0.16-4.9) | 0.89 |  |  | 15 | 1 | **0.07** | 7 (0.6-81.69) | 0.13 | 7(0.6-81.69) | 0.13 |
| >40 | 40 | 10 | **0.25** | Ref. |  |  |  | 40 | 16 | **0.40** | Ref. |  |  |  |
| **Gender** |  |  |  |  |  |  |  |  |  |  |  |  |  |  |
| Female | 20 | 5 | **0.25** | Ref. |  |  |  | 37 | 12 | **0.32** |  |  |  |  |
| Male | 71 | 21 | **0.30** | 1.26 (0.41-3.92) | 0.69 |  |  | 27 | 8 | **0.30** | 0.88 (0.3-2.58) | 0.82 |  |  |
| **Presence of BCG Scar** |  |  |  |  |  |  |  |  |  |  |  |  |  |  |
| No | 74 | 19 | **0.26** | Ref. |  |  |  | 50 | 14 | **0.28** | Ref. |  |  |  |
| Yes | 17 | 7 | **0.41** | 2.03 (0.68-6.08) | 0.21 |  |  | 14 | 6 | **0.43** | 1.93 (0.57-6.57) | 0.3 |  |  |
| **Duration of cough of index, wks.** |  |  |  |  |  |  |  |  |  |  |  |  |  |  |
| 1-4 | 15 | 5 | **0.33** | 1.96 (0.57-6.82) | 0.3 |  |  | 37 | 10 | **0.27** | 2.37 (0.68-8.23) | 0.18 |  |  |
| 5-10 | 59 | 12 | **0.20** | Ref |  |  |  | 15 | 7 | **0.47** | Ref |  |  |  |
| ˃10 | 17 | 9 | **0.53** | 4.41(1.41-13.84) | **0.02** |  |  | 12 | 3 | **0.25** | 0.9 (0.21-4.02) | 0.89 |  |  |
| **Time Spent with index, hrs.** |  |  |  |  |  |  |  |  |  |  |  |  |  |  |
| 2-12 | 23 | 4 | **0.17** | Ref. |  |  |  | 37 | 11 | **0.30** | Ref. |  |  |  |
| >=13 | 68 | 22 | **0.32** | 2.28 (0.69-7.49) | 0.18 |  |  | 27 | 9 | **0.33** | 1.19 (0.41-3.44) | 0.76 |  |  |
| **Number of household occupants** |  |  |  |  |  |  |  |  |  |  |  |  |  |  |
| 1-5 | 55 | 13 | **0.24** | Ref. |  |  |  | 30 | 8 | **0.27** | Ref. |  |  |  |
| 6-10 | 30 | 10 | **0.33** | 1.62 (0.61-4.32) | 0.34 |  |  | 26 | 8 | **0.31** | 1.23 (0.39-3.91) | 0.74 |  |  |
| >10 | 6 | 3 | **0.50** | 3.24 (0.59-17.99) | 0.19 |  |  | 8 | 4 | **0.50** | 2.75 (0.56-13.69) | 0.22 |  |  |
| **Household structure** |  |  |  |  |  |  |  |  |  |  |  |  |  |  |
| Shack | 33 | 6 | **0.18** | Ref. |  |  |  | 17 | 5 | **0.29** | Ref. |  |  |  |
| Brick | 58 | 20 | **0.34** | 2.37 (0.84-6.69) | 0.11 |  |  | 47 | 15 | **0.32** | 1.13 (0.34-3.78) | 0.85 |  |  |
| **Number of house windows** |  |  |  |  |  |  |  |  |  |  |  |  |  |  |
| 0-3 | 34 | 4 | **0.12** | Ref. |  |  |  | 21 | 5 | **0.24** | Ref. |  |  |  |
| 4-6 | 27 | 9 | **0.33** | 3.75 (1.01-13.97) | 0.05 |  |  | 21 | 7 | **0.33** | 1.6 (0.42-6.2) | 0.5 |  |  |
| ≥7 | 30 | 13 | **0.43** | 5.74 (1.62-20.4) | 0.01 |  |  | 22 | 8 | **0.36** | 1.83 (0.49-6.9) | 0.38 |  |  |
| **Household habitable rooms** |  |  |  |  |  |  |  |  |  |  |  |  |  |  |
| 1-3 | 36 | 3 | **0.08** | Ref. |  | Ref. |  | 20 | 4 | **0.20** | Ref. |  |  |  |
| >3 | 55 | 23 | **0.42** | 7.91 (2.16-28.95) | **0.01** | 7.91  (2.16-28.94) | **0.002** | 44 | 16 | **0.36** | 2.29 (0.66-8.03) | 0.2 |  |  |
| **Relationship to index case** | |  |  |  |  |  |  |  |  |  |  |  |  |  |
| Spouse/child | 43 | 7 | **0.16** | Ref. | Ref |  |  | 11 | 3 | **0.27** | Ref. | Ref |  |  |
| Other/Non relative | 48 | 19 | **0.39** | 3.36 (1.25-9.11) | **0.02** |  |  | 53 | 17 | **0.32** | 1.26 (0.3-5.38) | 0.76 |  |  |

Reconstruction and Development Programme (RDP), All variables in this model were included without predefined categorization as independent or adjustment variables. As such, the reported estimates should not be interpreted as causal effects but rather as statistical associations that warrant further investigation
